# A Device for Performing Automated Balloon Catheter Inflation Ischemia Studies

**DOI:** 10.1371/journal.pone.0095823

**Published:** 2014-04-25

**Authors:** Silas J. Leavesley, Whitley Ledkins, Petra Rocic

**Affiliations:** 1 Chemical and Biomolecular Engineering, University of South Alabama, Mobile, Alabama, United States of America; 2 Pharmacology, University of South Alabama, Mobile, Alabama, United States of America; 3 Center for Lung Biology, University of South Alabama, Mobile, Alabama, United States of America; 4 Pharmacology, New York Medical College, Valhalla, New York, United States of America; University of Otago, New Zealand

## Abstract

Coronary collateral growth (arteriogenesis) is a physiological adaptive response to transient and repetitive occlusion of major coronary arteries in which small arterioles (native collaterals) with minimal to no blood flow remodel into larger conduit arteries capable of supplying adequate perfusion to tissue distal to the site of occlusion. The ability to reliably and reproducibly mimic transient, repetitive coronary artery occlusion (ischemia) in animal models is critical to the development of therapies to restore coronary collateral development in type II diabetes and the metabolic syndrome. Current animal models for repetitive coronary artery occlusion implement a pneumatic occluder (balloon) that is secured onto the surface of the heart with the suture, which is inflated manually, via a catheter connected to syringe, to effect occlusion of the left anterior descending coronary artery (LAD). This method, although effective, presents complications in terms of reproducibility and practicality. To address these limitations, we have designed a device for automated, transient inflation of balloon catheters in coronary artery occlusion models. This device allows repeated, consistent inflation (to either specified pressure or volume) and the capability for implementing very complex, month-long protocols. This system has significantly increased the reproducibility of coronary collateral growth studies in our laboratory, resulting in a significant decrease in the numbers of animals needed to complete each study while relieving laboratory personnel from the burden of extra working hours and enabling us to continue studies over periods when we previously could not. In this paper, we present all details necessary for construction and operation of the inflator. In addition, all of the components for this device are commercially available and economical (Table S1). It is our hope that the adoption of automated balloon catheter inflation protocols will improve the experimental reliability of transient ischemia studies at many research institutions.

## Introduction

Type II diabetes and the metabolic syndrome – a cluster of risk factors including abdominal obesity, insulin resistance, hyperglycemia, dyslipidemia and hypertension – affect ∼30% of the U.S. population with increasing prevalence [1]. These pathologies are also associated with increased severity of ischemic coronary artery disease (CAD). For example, higher numbers of metabolic syndrome components have been correlated with more severe CAD [1], [2] and increased CAD-associated mortality. Patients with type II diabetes are ∼2 times more likely to die of CAD, whereas patients with all component pathologies of the metabolic syndrome are ∼3.6–4.4 times more likely to die of CAD [3], [4]. Moreover, current revascularization therapies, coronary artery bypass grafting (CABG), and percutaneous transluminal coronary angioplasty (PTCA) in type II diabetics and metabolic syndrome patients are associated with higher procedural risk and poorer long-term outcomes then in patients without type II diabetes or the metabolic syndrome [5]–[7].

Coronary collateral growth (arteriogenesis) is a physiological adaptive response to transient and repetitive occlusion of major coronary arteries in which small arterioles (native collaterals) with minimal to no blood flow remodel into larger conduit arteries capable of supplying adequate perfusion to tissue distal to the site of occlusion. Transient repetitive coronary artery occlusion and resultant myocardial ischemia stimulate coronary collateral growth in healthy humans and normal animals [8]–[11]. Clinically, patients with stable angina have decreased incidence of fatal myocardial infarction, which is associated with better developed collateral networks [8]. In addition, well-developed collateral networks seem to promote long term patency of coronary bypass grafts. However, this normal physiological response is impaired in patients with type II diabetes and the metabolic syndrome [12]–[15]. Graft closure, and consequent need for revascularization, is a significant problem in type II diabetic and metabolic syndrome patients [6]. Therefore, the ability to reliably and reproducibly mimic transient, repetitive coronary artery ischemia in animal models is critical to the development of therapies to restore coronary collateral development in type II diabetes and the metabolic syndrome.

Like in human metabolic syndrome, coronary collateral growth has been shown to be impaired in most animal models of the metabolic syndrome [10], [16]–[18]. However, normal collateral development has been reported in a swine model of the metabolic syndrome [19]. The most obvious difference in the swine model is that this was a model of progressive chronic ischemia whereas the other animal models used transient, repetitive coronary artery occlusion to stimulate collateral development, which mimics the pathophysiology of the human. Since the exact timing and duration of coronary occlusions has been associated with the extent of collateral growth [20], [21], the difference between these two methods of inducing coronary occlusion is a likely explanation for the different outcomes between the models and emphasizes the necessity of using transient and repetitive coronary occlusion models vs. progressive occlusion (ameroid constrictors) when studying coronary collateral development.

We and others have recently successfully used both normal and metabolic syndrome rat models of transient, repetitive left anterior descending coronary artery (LAD) occlusion to study coronary collateral development [10], [16], [18], [22]–[25]. A pneumatic occluder (balloon) is secured onto the surface of the heart with the suture, which is also passed under the LAD so that when the balloon is inflated, the LAD is occluded [23]. The occluder has a catheter, which is externalized for easy access for manual inflation, using an air-filled syringe, post-surgically. This method, although effective, presents complications in terms of reproducibility and practicality. Most significantly, manual inflation allows for regulation of the volume of air injected into the occluder but provides no indication of consistent pressure from animal to animal. Since occluders are manufactured individually and manually, some variation in their size is inevitable. Therefore, despite identical volumes being injected, the varying inflation pressure and consequent varying extent of LAD occlusion introduces a small, yet unnecessary confounding variable into the experimental design. Secondly, it can be difficult to maintain a stringent schedule demanded by manual occlusion inflation protocols for periods of several weeks or months, especially in smaller laboratories. Because the timing and duration of transient coronary occlusions has been associated with the extent of collateral growth (as mentioned above), these small variations in LAD occlusion or frequency may also effect variations in the extent of collateral growth.

To overcome these obstacles, we have developed an automated inflation system. This system allows reproducible pressurized inflation of up to four pneumatic occluders simultaneously. In addition, complex and varied experimental protocols can be defined including inflation times, repetition rates, resting times, and the number of cycles for each occlusion protocol (of up to indefinite duration). This system has significantly increased the reproducibility of coronary collateral growth studies in our laboratory, resulting in a significant decrease in the numbers of animals needed to complete each study while relieving laboratory personnel from the burden of extra working hours and enabling us to continue studies over periods when we previously could not.

In this paper, we describe the construction and operation of a device for automated inflation of pneumatic occluders in transient LAD ischemia studies. The device consists of electronics and pneumatics hardware, and corresponding control software. Each element of the device is described in detail, so as to allow construction of similar devices at other research institutions. In addition, a detailed list of all components, with supplier, and description of the operating software are included in the supplemental information. All of the components for this device are commercially available, with a total cost of under $1300 (U.S., not including computer). It is our hope that the adoption of automated balloon catheter inflation protocols will improve the experimental reliability of transient ischemia studies at many research institutions.

## Materials and Methods

The automated balloon catheter inflation device consisted of an electronic subsystem, an pneumatic subsystem, and control software with corresponding user interface. The control software communicated with the electronic subsystem via an USB interface board. The electronic subsystem supplied power to solenoid pressure valves, which controlled the pressurizing and depressurizing of the balloon catheters. A list of the parts required for the automated catheter inflation device is given in Table S1.

### Electronic Subsystem

**Figure 1:**
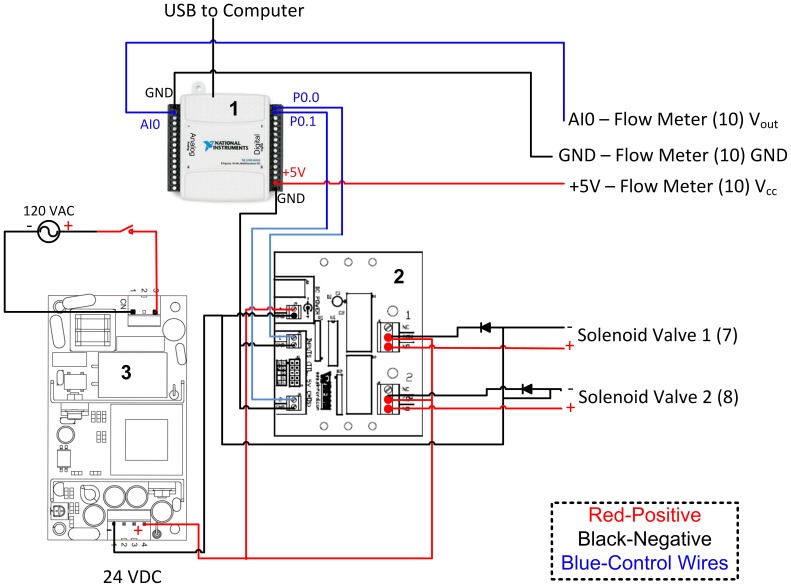
Electronic subsystem schematic. Red lines indicate positive voltage supply (+), black lines indicate negative (−), and blue lines indicate control (digital TTL or analog 0–5 V). Component images for the USB interface (1), relay board (2), and 24 VDC power supply (3), are taken from National Instruments [28], CUI [29], and Winford Engineering [30], respectively.

The electronic subsystem consisted of an USB interface (USB-6009, National Instruments, Inc.), 24V DC power supply (VOF-25-24, CUI, Inc.), and TTL-controlled 24V relay board (RLY102-24V, Winford Engineering), along with supporting electrical components (Figure 1). Digital input/output lines from the USB interface were used to switch the relays, controlling 24 V power to the solenoid valves. A snubber circuit was used for each solenoid, consisting of a diode connected in reverse polarity from the negative terminal of the solenoid to the positive DC voltage supply. This circuit protects against high reverse potentials generated from solenoid closing. AC Power to the 24 V supply (and hence, the relay board) was controlled by a manual switch.

An air flow meter (D6F-01A1-110, Omron) was used to measure the volume of air delivered. The flow meter output signal was read via an analog input (0–5 V, 14 bit) and integrated using a Labview script (see Control Software section).

All components for the electronic subsystem were placed in a water-tight enclosure. Water-resistant electrical fittings were used for the solenoid valve and flow meter connections.

### Pneumatic Subsystem

**Figure 2:**
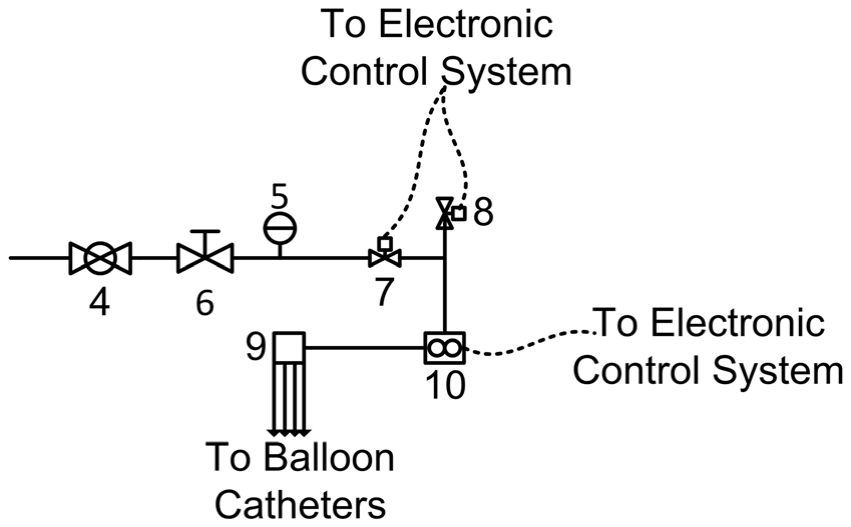
Pneumatic subsystem schematic: main pressure valve (4), pressure dial (5), pressure regulator (6), pressurizing solenoid valve (7), venting solenoid valve (8), pressure manifold (9), and air flow meter (10). Solid lines indicate pneumatic connections. Dashed lines indicate electrical connections (see Figure 1).

The pneumatic subsystem consisted of a master pressure control valve, two solenoid valves, the flow meter, and the pressure manifold (Figure 2). Using the dual relay, the two solenoid valves could be operated independently. Three valve combinations were used for catheter inflation: 1) the first solenoid valve (component (7) in Figure 2) was opened and the second solenoid valve (component (8) in Figure 2) was closed, allowing pressurized air to flow into the catheters; 2) the first and second solenoid valves were both closed, maintaining a fixed pressure within the catheters balloons, 3) the first solenoid valve was closed and the second solenoid valve was opened, allowing pressurized air to vent to the atmosphere. Valves were kept in the third configuration between inflation cycles, allowing balloon catheters to remain deflated.

### Control Software

Control software was written using Labview software (National Instruments, Inc.). The control software consisted of a series of timed loops, implementing a series of balloon inflation and deflation cycles (see Figure S1 for a detailed description). A user interface was also generated using Labview software, in order to facilitate changing the inflation protocol for different experiments.

The factory calibration for the flowmeter was also applied using the control software, using a signal scaling function. The calibrated signal was then integrated to yield a measure of the total air volume delivered. The exact Labview functions for performing these steps are shown in Figure S1.

### Comparison of Manual and Automated Balloon Catheter Inflation

Variation in collateral dependent blood flow in rat repetitive myocardial ischemia (RI) studies was used as an indicator of balloon catheter inflation reproducibility. Data was tabulated from both unpublished and published [23]–[26] results from our group. Inclusion criteria for the retrospective comparison included: matching the mouse line (different breeds may introduce inherit physiological variance), matching the study protocol, and matching (as closely as possible) the sample size for the study. Two of the more recent automated inflation experiments performed by our group [24], [27] were excluded, as these studies involved much smaller sample sizes than the manual inflation studies (n = 5 vs. n = 12, respectively). Blood flow measurements were performed using either radioactive microspheres or gold-labeled and samarium-labeled microspheres, as described previously [23], [25] (for unpublished results, the methodology is described in detail in the following sub-sections). Data from two rat lines was used for comparison: Wistar Kyoto (WKY) rats and Sprague-Dawley (SD) rats. For both rat lines, collateral dependent blood flow measurements were made either before and after RI or in RI-affected collateral zone (CZ) and normal zone (NZ) tissues. All measurements were made using both manual inflation and the automated balloon catheter inflation device described in these studies.

#### Rat model of coronary collateral growth (CCG)/repetitive ischemia (RI)

Male, 10–12 week old Sprague-Dawley (SD; Charles Rivers, Wilmington, MA) (300–350 g) were used for chronic (9 days) implantation of a pneumatic occluder over the left anterior descending coronary artery (LAD). A suture was passed under the proximal portion of the LAD and the occluder was sown onto the surface of the heart. The occluder catheter was externalized between the scapulae. When the occluder is inflated, the suture is pulled towards the surface of the heart and the LAD is occluded. The LAD perfusion territory is termed the collateral-dependent zone (CZ) because perfusion in this area, while the LAD is occluded, depends on the development of coronary collaterals. The animals underwent a RI protocol consisting of eight 40 sec occlusions, once every 20 min (2 h, 20 min total) followed by a rest period of 5 h, 40 min. This 8-hour cycle was repeated 3 times/day for 0–9 days. Surgical procedures were performed in accordance with the Animal Welfare Act and are approved by the IACUC of the University of South Alabama.

#### Myocardial and collateral-dependent blood flow measurements

Color microspheres (5×10^5^, 15 µM diameter) labeled with samarium (day 0 RI (initial surgery) or gold (day 10 RI) or radioactive microspheres (5×10^5^, 15 µM diameter) labeled with ^57^Co (day 0 RI (initial surgery) or ^103^Ru (day 10 RI) were injected into the LV during LAD occlusion. There are no differences in blood flows measured by colored or radioactive microspheres. Arterial reference blood samples (carotid) and heart tissue from the NZ and the CZ were collected, weighed and sent to BioPal (Worcester, MA) for analysis. Blood flows to the NZ and the CZ (ml/min/g) were calculated from the formula: Blood Flow = [(radioactive counts in myocardial tissue)X(blood reference withdrawal rate)/(radioactive count in blood reference)]/(weight of myocardial tissue). Results were analyzed by calculating the standard error of the mean (SEM):
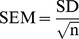
(1)where SD is the standard deviation and n is the number of samples. The coefficient of variation (CV) was then calculated:
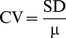
(2)where μ is the mean value. The CV was used as a metric to characterize the reproducibility of each study evaluated.

## Results

### System Description

**Figure 3:**
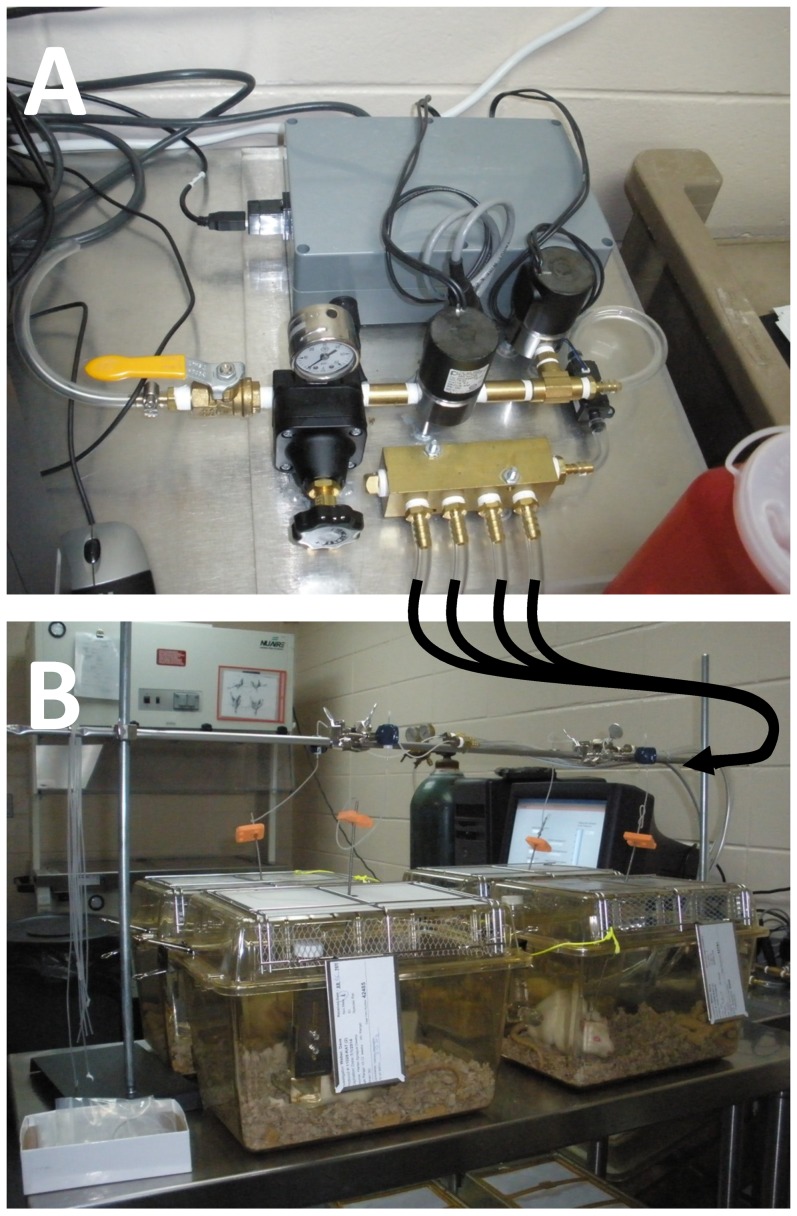
The automated catheter inflation device (A) and example of cardiac ischemia study using the device (B). The curved arrows indicate the pressure lines which exit from the manifold in (A) and are connected to the implanted balloon catheters in (B) via rotating couplers.

**Figure 4:**
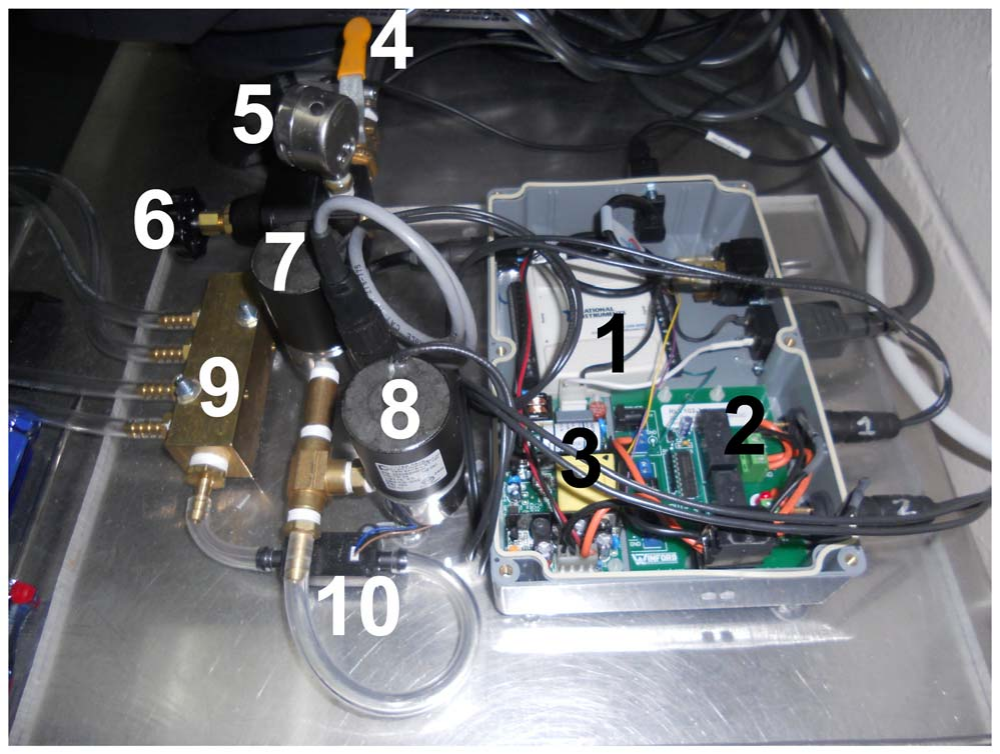
The completed balloon catheter inflation device consists of: the USB interface (1), relay board (2), 24 VDC power supply (3), main pressure valve (4), pressure dial (5), pressure regulator (6), pressurizing solenoid valve (7), venting solenoid valve (8), pressure manifold (9), and air flow meter (10). Components 1–3 comprise the electronic system while components 4–10 comprise the pneumatic subsystem.

**Figure 5:**
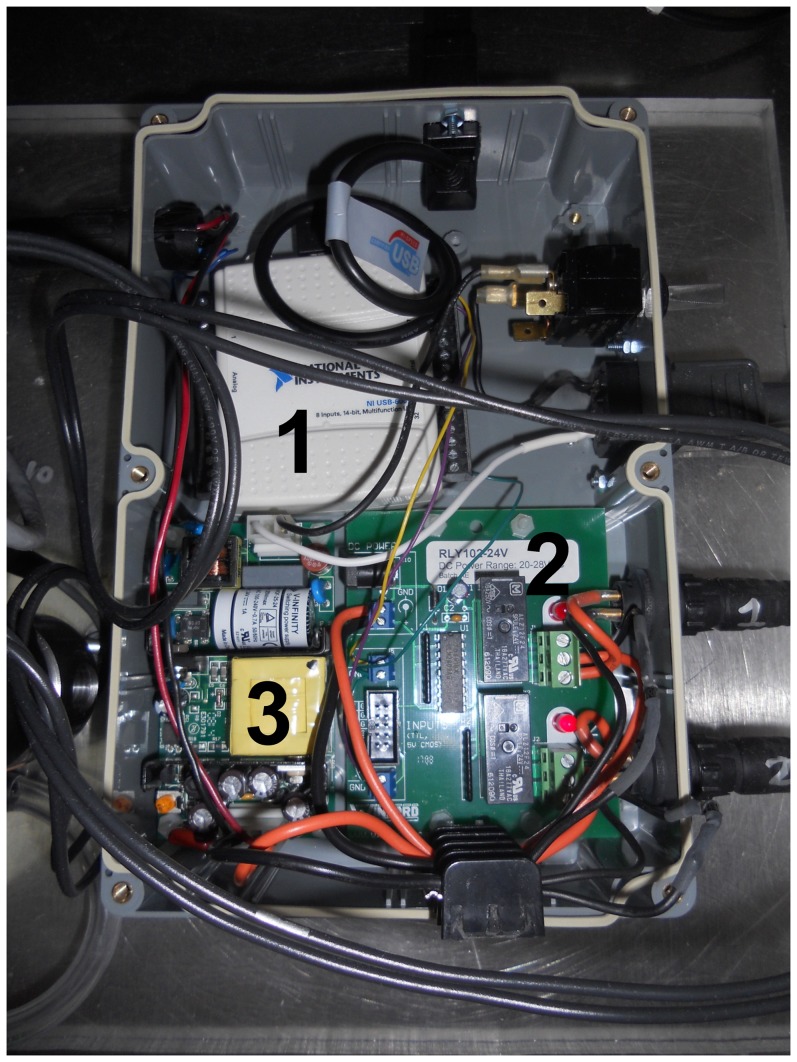
Detailed photograph of the electronics control system: the USB interface (1), relay board (2), and 24 VDC power supply (3).

The automated catheter inflation device provided consistent inflation volumes and balloon pressures. The manifold allowed up to four catheters to be inflated and deflated simultaneously (Figure 3). All components were integrated on a 2 ft×2 ft piece of Plexiglas for easy transport (Figure 4). 120 VAC and 24 VDC wire connections were enclosed in appropriate connectors or solder connections were enclosed in heat-shrink tubing to minimize the risk for short circuits. The electronic subsystem was placed in a water-tight enclosure (Figure 5).

**Figure 6:**
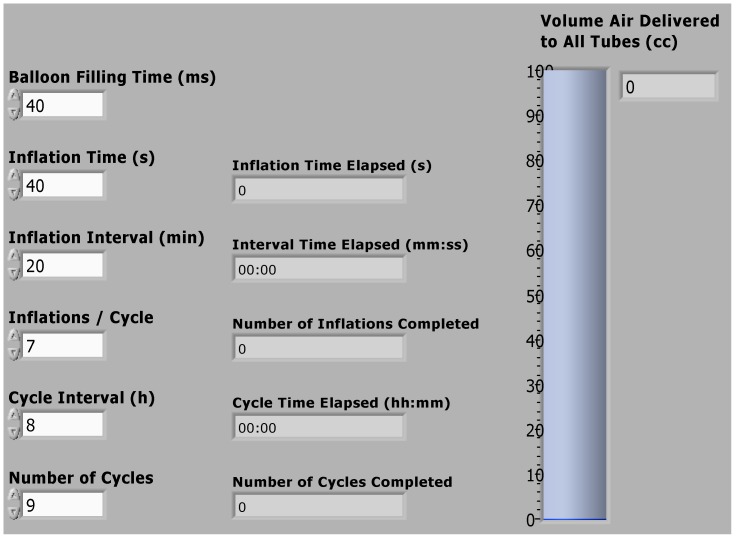
The software user interface (Labview front panel) allows the user to adjust parameters for automated inflation: the catheter pressurization (balloon filling time); the duration that the catheter remains inflated (inflation time); the interval between subsequent, shortly-spaced inflations in a cycle (inflation interval); the number of inflations per cycle (inflations/cycle); the interval between cycles (cycle interval); and the total number of cycles. For each of these sequences, a timer or counter displays the progress of the automated inflation. A bar plot displays the inflation volume, as measured by the flow meter.

The user interface allowed relatively complex or lengthy experimental protocols to be performed automatically – up to several weeks in duration (Figure 6). The user interface also allowed for monitoring of the protocol progress through a series of counters and timers. The software was compiled into an executable file (with a corresponding installer) that could be run on any windows-based computer.

### Comparison of Manual and Automated Balloon Catheter Inflation

**Figure 7:**
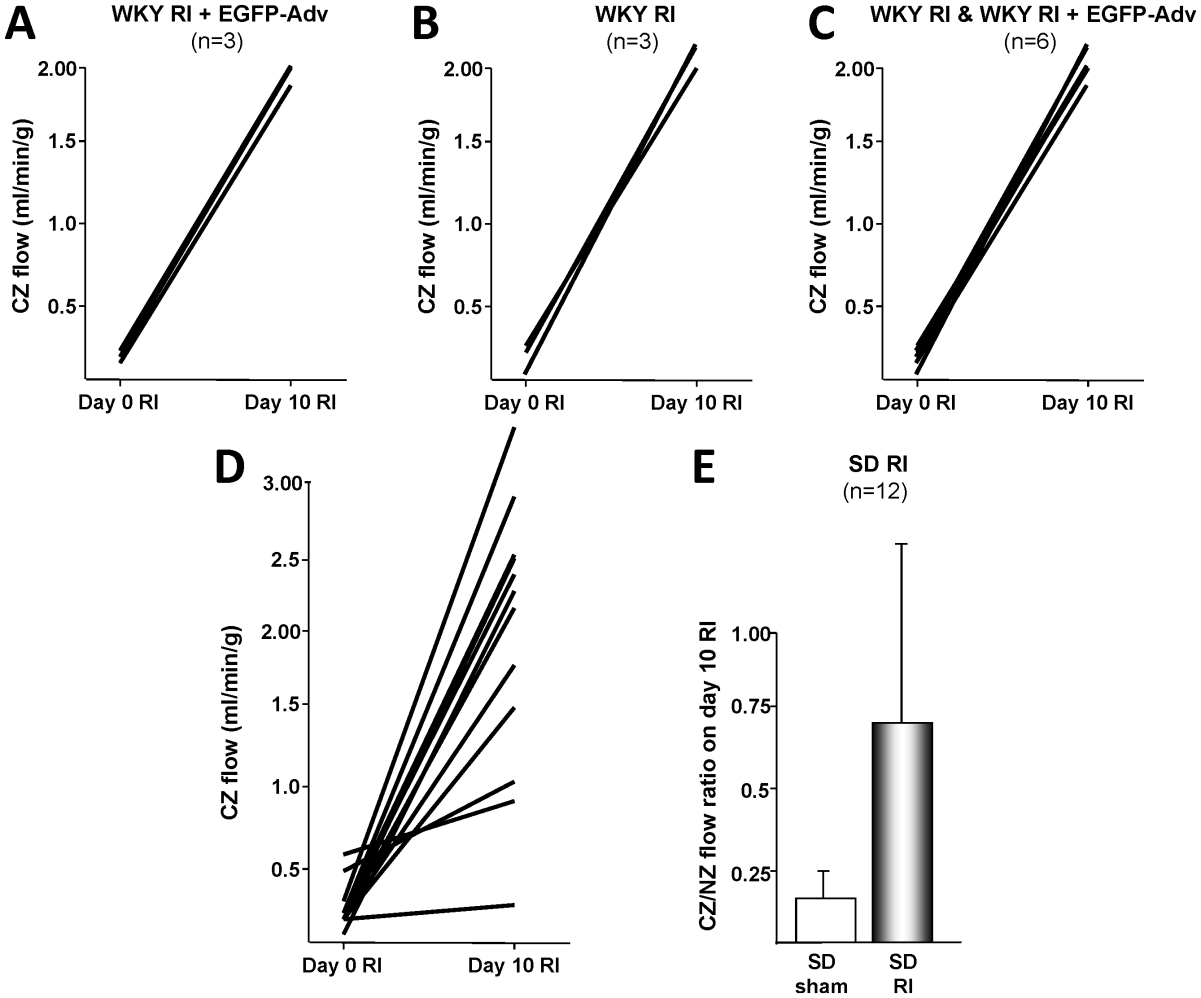
Collateral dependent blood flow measurements made after transient repetitive myocardia ischemia (RI) studies using the automated balloon catheter inflation device described in this manuscript displayed greatly reduced variance compared to blood flow measurements made after transient ischemia using manual inflation. Blood flow measurements made in WKY rats after using the automated inflation device (A–C) not only displayed greatly reduced variance than our previous manual inflation studies (compare to Figure 2 in Toyota, et al., 2005) [23], but also required fewer animals to complete a similar study (n = 3 or n = 6, compared to n = 9, for a study group). Note that panel C represents data from panels A and B combined. Similarly, blood flow measurements made in SD rats after manual inflation (D, E) had such high variance that they were not statistically significant (even with an n = 12), while measurements made after automated inflation (compare to Figure 7 in Dodd, et al., 2011 and Figure 4C in Hutcheson, et al., 2013) [24] achieved a high level of significance (p<0.05) with fewer animals (n = 5 and n = 7, respectively). A summary of results with coefficients of variation is given in Table 1.

One of the central outcomes of automated balloon catheter inflation is to ensure reproducible occlusion during repetitive myocardial ischemia (RI) studies. A key outcome of these studies is the development of coronary collateral growth, which can be assessed through the measurement of collateral dependent blood flow. Hence, variation in collateral dependent flow among animals in the same study group is one of the most important indicators of the reproducibility of balloon catheter inflation experiments. We have previously performed manual inflation protocols in WKY rats that resulted in collateral dependent blood flows of 0.26±0.09 mL/min/g (± indicates standard error of the mean (SEM); coefficient of variation (CV) = 1.038) and 1.63±0.3 (CV = 0.552) for pre- and post-RI, respectively (n = 9 animals) [23]. We have more recently performed these same protocols using the automated inflation device described in this paper, resulting in collateral dependent blood flows of 0.15±0.03 (CV = 0.470) and 2.04±0.05 (CV = 0.064) for pre- and post-RI, respectively (n = 6, see Figure 7, A–C for graphical representation) [25]. Similar studies were attempted using Sprague-Dawley rats, where manual studies resulted in highly variant blood flows (CV = 0.523 and 0.463) that were not publishable, even with a large sample size (n = 12, Figure 7, D). By comparison, automated inflation studies provided blood flows with reduced variation (CV = 0.039 and 0.067) and statistically-significant results, while requiring fewer animals (n = 7) [26]. A summary of the multi-study comparison is presented in Table 1.

**Table 1:** Summary of collateral dependent blood flow measurements made using manual and automated inflation protocols for before and after transient repetitive myocardia ischemia (RI).

	Manual Inflation				Automated Inflation			
Study Group	Collateral Dependent Blood Flow (mL/min/g)	SEM	CV	n	Collateral Dependent Blood Flow (mL/min/g)	SEM	CV	n
**WKY**	0.26	0.09	1.038	9	0.15	0.03	0.470	6
**WKY RI**	1.63	0.3	0.552	9	2.04	0.05	0.064	6
**SD**	0.25	0.04	0.523	12	2.33*	0.03	0.039	7
**SD RI**	1.97	0.26	0.463	12	1.95	0.05	0.067	7

Automated inflation produced a marked decrease in the standard error of the mean (SEM) and coefficient of variation (CV), even with reduced sample numbers (n). Data were tabulated from several studies for this comparison: WKY manual inflation studies [23], WKY automated inflation studies [25], SD manual inflation studies (unpublished), SD automated inflation studies [26].

*Indicates normal-zone blood flow (not collateral dependent flow) measured at 0 days RI.

## Discussion

Balloon catheter inflation experiments have been vital for simulating myocardial ischemia in animal models [16], [18], [23]. In these approaches, a catheter balloon is inflated to reduce or eliminate blood flow to a desired region. Manual approaches for balloon catheter inflation have been successfully used in previous studies. However, we have found that manual balloon inflation requires intermittent-to-constant supervision, depending on the type of inflation protocol used. As shown (Table 1), the common method for supplying pressurized air – through a hand-held syringe – introduces a non-negligible amount of human error into the inflation protocol, resulting in higher variance in subsequent collateral dependent blood flow measurements, with coefficients of variation (CV) of 1 or greater. Also, some aspects of manual balloon catheter inflation (such as the rate of inflation and deflation) are operator dependent, and hence, difficult to reproduce in varied settings. Because of all of these reasons, we have designed the automated balloon catheter inflation device reported here, which reduces the need for operator supervision, increases the reproducibility of balloon inflation, and allows multi-week experimental protocols to be performed. We have controlled the balloon catheter inflation device with a software package that allows for easy set-up and alteration of the inflation protocol, as well as monitoring of the progress of the inflation protocol and an estimate of the volume of pressurized air delivered to the balloons. This device has resulted in reduced variation in collateral dependent blood flow measurements. The highest CV reported with automated inflation was 0.470, which was measured at conditions of very low blood flow rate (likely close to the instrumentation error of the flow measurement technique). All other CVs for automated inflation were <0.1. By contrast, CVs for manual inflation ranged from 0.436–1.038. It should also be noted that, in both WKY and SD rats, fewer animals were required for each study using automated inflation, while still yielding blood flow measurements with decreased CVs.

The automated balloon catheter inflation device that we have developed provides several significant advantages over previous manual inflation studies. First, collateral dependent blood flow measurements made after using automated inflation have greatly reduced variance when compared to those made after manual inflation (Figure 7 and Table 1). Second, this reduced variance has allowed us to reduce the number of animals needed to achieve statistically-significant results, and hence has allowed a decreased morbidity (mortality) rate associated with transient ischemia studies. Third, automated inflation has significantly reduced the number of man-hours required to complete an animal study – both due to the need for less monitoring and interaction with animals and due to a fewer number of animals needed. Finally, we anticipate that automated balloon catheter inflation greatly reduces the potential for over-inflation of the balloon, which may serve to better preserve the mechanical integrity of the balloon. Consistent mechanical integrity may, in turn, be one of the factors leading to reduced variance among animals with automated balloon catheter inflation. While beyond the scope of this study, the integrity of the balloon catheter could be assessed using stress-strain testing and a load failure analysis.

The automated balloon catheter inflation device costs roughly $1,200–$2,000 (US) to build, depending on the availability of parts (listed in Table S1) and technical support. Hence, this represents a very economical investment, when compared to the number of man-hours required for a single, multi-week catheter inflation protocol (which is usually repeated multiple times for statistical significance). Although routine monitoring of any animal protocol is necessary, using an automated inflation device reduced the need for human supervision from hourly to daily. In addition, the dependence of the accuracy of the protocol on operator interaction was largely eliminated.

The volumetric flowrate that was measured (by integrating the flowmeter signal) represents an estimate of the total volume delivered to all four balloon catheters. However, because of changes in pressure, and because of the compliance of catheter lines and connecting tubing, this measured volume was not interpreted as an absolute volume of air delivered, but was rather used to verify the reproducibility of the device. Because the balloon catheters are pressurized in parallel, it should also be noted that the volume expansion of each balloon catheter may vary, depending on manufacturing differences in the balloon. In our previous work, we have seen significant manufacturing variation in balloon volume. Hence, prior to beginning an animal protocol, it is important to test each of the balloon catheters used. The automated inflation device we have developed also aids in this pre-experimental testing, a multiple balloons can be simultaneously inflated and their volumes compared. In addition, a series of rapid inflation/deflation cycles can be run to check for defects in the balloons or catheter lines. It should also be noted that this device can easily be modified to allow connection to more than four catheter lines by replacing the pressure manifold with a manifold having more ports.

The automated inflation device was controlled through a custom Labview program with user interface. This program was compiled as an executable, to allow easy access by users and to allow the program to be installed on multiple PCs. However, it should be noted that the executable still requires a deployable version of Labview with associated virtual instrument libraries. Because of this, there is some amount of computational overhead required to run the automated inflation device software. If very high speed inflation/deflation cycles were required (on the order of µs-ms), it would be advisable to develop a stand-alone version of the software, that did not require the overhead of the deployable version of Labview. However, for the time scales of most studies (inflation times of seconds), the Labview-based software we have developed provides more-than-adequate response speed and reproducibility.

## Conclusion

The ability to repetitively occlude the coronary artery is critical to the use of animal models for transient coronary ischemia. In our previous work, we have found that repeated occlusion of the coronary artery through manual inflation of balloon catheters can achieve reproducible changes in collateral dependent blood flow. However, relatively large numbers of animals and man-hours were often required to achieve statistical significance, and a high level of variance was often noted among study animals. In this work, we have developed a device for repetitive automated inflation of balloon catheters. We have also compared manual and automated inflation studies and shown that studies using this automated inflation device have demonstrated greatly reduced variance, reduced need for animals, and reduced man-hours to complete a given study. We have fully documented the parts, equipment, procedures, and software needed, enabling the automated inflation device to be constructed by technical or engineering staff at most institutions. Finally, in addition to the reduced variance, reduced need for animals, and reduced man-hours required to complete a study, adoption of automated inflation protocols using this (or similar) device should result in enhanced data quality in the field of transient ischemia studies and a better ability to compare results amongst research groups.

## Supporting Information

Figure S1 Labview back panel. The main portion of the software consists of a series of “filmstrip” timing loops, indicated by the thick lines with squares.
(TIF)

Table S1 Table of parts required to build the automated balloon catheter inflator.
(DOCX)
