# Supplementary figures and images for: A Device for Performing Automated Balloon Catheter Inflation Ischemia Studies
Source: PLoS One. 2014 Apr 25;9(4):e95823. doi: 10.1371/journal.pone.0095823 (PMC4000226; doi:10.1371/journal.pone.0095823)

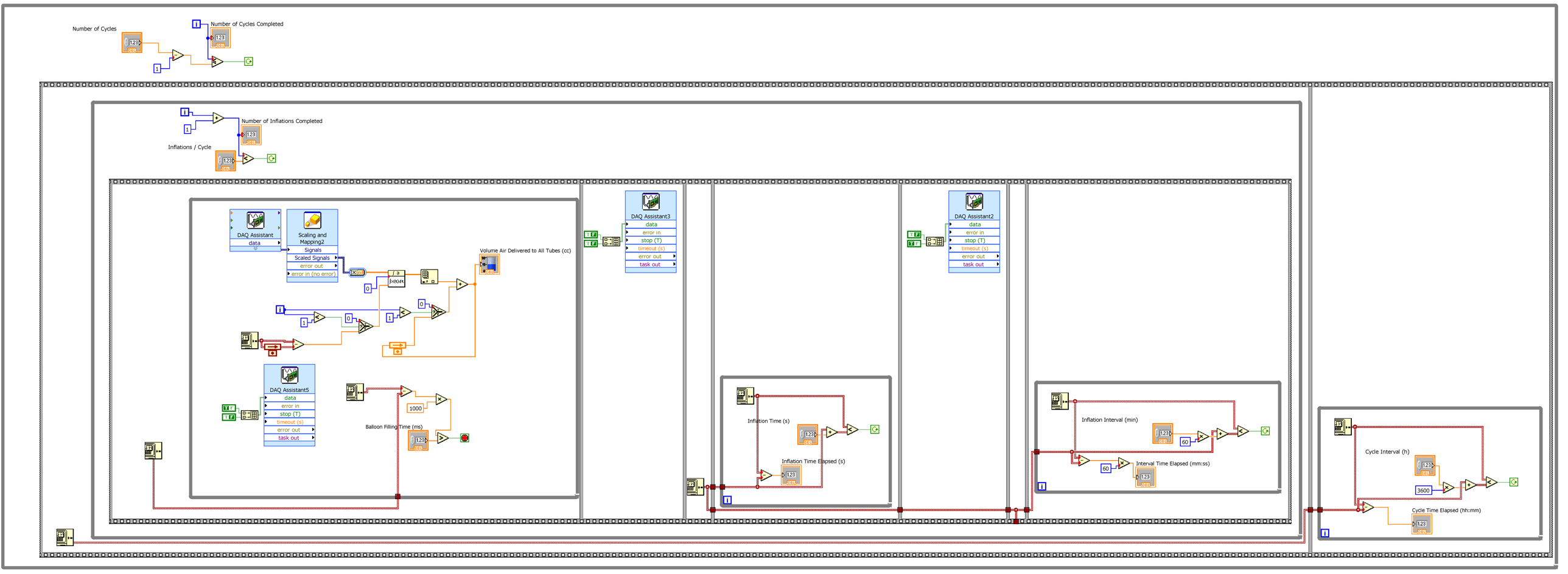

Supplement: Figure S1 — Labview back panel. The main portion of the software consists of a series of “filmstrip” timing loops, indicated by the thick lines with squares. (TIF) [file pone.0095823.s001.tif]
