# Supplementary material for: A Device for Performing Automated Balloon Catheter Inflation Ischemia Studies
Source: PLoS One. 2014 Apr 25;9(4):e95823. doi: 10.1371/journal.pone.0095823 (PMC4000226; doi:10.1371/journal.pone.0095823)
Supplement: Table S1 — Table of parts required to build the automated balloon catheter inflator. (DOCX) [file pone.0095823.s002.docx]

| **Description** | **Supplier Part Number** | **Supplier** | **Quantity** |
| --- | --- | --- | --- |
| USB Analog and Digital Control Board | USB-6009 | National Instruments | 1 |
| 24V Power Supply (1.0 A – 24 W) | 102-1328-ND | Digikey | 1 |
| 24V Dual DC Power Relay | RLY102-24V | Winford Engineering | 1 |
| Air Flow Sensor, Type 0-1LTR PLST | Z1671-ND | Digikey | 1 |
| 24"x24"x0.5" Plexiglass | 44369 | US Plastics | 1 |
| Standard-Wall Brass Threaded Pipe Nipples and Pipe (threaded on both ends): 1/4" pipe size by 1.5" pipe | 4568K132 | McMaster-Carr | 4 |
| Low-Pressure Brass Threaded Pipe Fittings: 1/4" tee with female threads | 4429K251 | McMaster-Carr | 1 |
| Brass Barbed Hose Fittings: 1/4" hose ID, 1/4" pipe size (10 pack) | 5346K14 | McMaster-Carr | 1 |
| Brass Barbed Hose Fittings: 1/4" hose ID, 3/8" pipe size (5 pack) | 5346K14 | McMaster-Carr | 1 |
| Air Regulators: 1/4" pipe size, 1 to 60 psi pressure range (regulator) | 41735K201 | McMaster-Carr | 1 |
| Air Regulators: 1/4" pipe size, 1 to 60 psi pressure range (mounting brackets) | 41735K48 | McMaster-Carr | 2 |
| Brass Ball Valve : 1/4" pipe size, lever | 47865K21 | McMaster-Carr | 1 |
| Glycerin Filled Gauge with NIST Certificate: 1/4" pipe size, 2.5" dial size, pressure gauge (0 to 60 psi pressure range), standard bottom connection mount | 3481K11 | McMaster-Carr | 1 |
| Brass Manifold: 4 outlets, 3/8" inlet pipe size, 1/4" outlet pipe size | 5627K512 | McMaster-Carr | 1 |
| Brass Manifold Hex-Head Brass Plug: 3/8" pipe size | 50785K23 | McMaster-Carr | 1 |
| L-com Connectivity ECF504-UAAS, Adapter; USB Type A - Type A A; USB; EMI/RFI | 70126195 | Allied Electronics | 1 |
| Switchcraft EN3C2M16X, CORD CONNECTOR, MALE, WEATHERTIGHT, #16CONTACTS, 2 PIN | 70214331 | Allied Electronics | 4 |
| Switchcraft EN3P2F16X, PANEL CONNECTOR, FEMALE, WEATHERTIGHT, #16 CONTACTS, 2 PIN | 70214360 | Allied Electronics | 4 |
| Switchcraft EN3P3FX, PANEL CONNECTOR, FEMALE, WEATHERTIGHT, #20 CONTACTS, 3 PIN | 70214365 | Allied Electronics | 4 |
| Switchcraft EN3C3MX, CORD CONNECTOR, MALE, WEATHERTIGHT, #20CONTACTS, 3 PIN | 70214349 | Allied Electronics | 6 |
| Switchcraft EN3C3FX, CORD CONNECTOR, FEMALE, WEATHERTIGHT, #20 CONTACTS, 3 PIN | 70214350 | Allied Electronics | 3 |
| Cherry Electrical WRG32F2FBBNN, Switch, Rocker, Power, DPST, On/Off, Black, Imprinted O/- | 70207352 | Allied Electronics | 1 |
| .156 TERMINAL HOUSING; W/LCK RAMP; 4 CIRCUITS | 70190664 | Allied Electronics | 30 |
| Double Angle USB 2.0 Cable /A to B: Down Angle A to Up Angle B: Length 5 in | RR-AR2BR4-05G (5 IN) | USBFirewire | 1 |
| Submersible SS Solenoid Valve Fluoroelastomer Seal, 1/4 NPT Fem, 250 PSI, 24 VDC, 1/4" pipe size, normally closed | 5077T133 | McMaster-Carr | 2 |
| Enclosure; Polyurethane Gasket; 11.024 in.x7.485 in.x3.85 in.; Stainless Screw | 70148568 | Allied Electronics | 1 |
| Electrical Connector: AC Male Power Inlet Connector, Flange, 120/250VAC, 15A (UL/CSA), 10A (VDE), UL94V-0 | 70186042 | Allied Electronics | 1 |
| RS S3 Rodent Swivel Stainless Steel, 21 gauge | RS S3 | Lomir Biomedical, Inc. | 4 |
| HI-Pressure Brass Single-Barbed Tube Fitting Adapter for 0.04" Tube ID X 1/4" NPT Female Pipe | 50745K42 | McMaster-Carr | 5 |
